# An immune surveillance model to monitor the response to neo-adjuvant chemotherapy treatment in breast cancer patients

**DOI:** 10.1186/2051-1426-3-S2-P261

**Published:** 2015-11-04

**Authors:** David A Bernal-Estévez, Oscar A García, Ramiro Sanchez, Carlos A Parra-López

**Affiliations:** 1 Fundación Salud de los Andes, Bogota, Colombia; 2 Instituto Nacional de Cancerología, Bogotá, Colombia; 3 Clínica del Seno, Bogotá, Colombia; 4 Universidad Nacional de Colombia, Bogota, Colombia

## Background

Tumors treated with doxorubicin generates immunogenic cell death of tumor cells, favoring cross-presentation of tumor antigens by DCs to CD8 T cells conferring antitumor immunity in vaccinated mice. On the other hand, low-dose of cyclophosphamide reduces the number of regulatory T cells (Treg). Although treatment with doxorubicin and cyclophosphamide (AC chemotherapy) is widely used in patients with breast cancer, the immune-stimulating effect of AC in these patients has been difficult to prove.

## Methodology

After signed informed consent, we compared in blood samples from breast cancer patients obtained pre- and post-chemotherapy several immunological parameters in order to monitor changes in the response of T and APC compartments during treatment. Using peripheral blood samples of patients collected before and after three cycles of AC we quantified by flow cytometry: (i) Tregs, (ii) MDSCs; (iii) myeloid and plasmacytoid DCs; (iv) the phenotype of Tumor Associated Antigens (TAAs) specific T cells; and (v) the T cell activation and DC maturation phenotype in response to TCR stimulation and to pro-inflammatory cytokines respectively.

## Results

We show a reversal of the suppression of T cell response to polyclonal stimuli and maturation of DCs in response to pro-inflammatory stimuli in samples from patients after three cycles of chemotherapy not observed in the pre-chemotherapy samples. Interestingly, in the same donor the T cell response (TCR internalization) highly correlates with IL-12 secretion by mature myeloid DC (CD83+). Using factor analysis and Principal Component Analysis (PCA), the data collected show significant differences between the three categories analyzed (pre- vs. post-chemotherapy patients and healthy donors).

## Conclusions

The analysis of the different variables by PCA lead us to argue for the capacity of chemotherapy to improve responsiveness of the T and APC compartments in treated patients to levels comparable to those of healthy controls that was not observed in samples from untreated patients. The application of this model of immune surveillance required a careful optimization process using small samples of blood and brief periods of in vitro stimulation. These interesting results must be validated with a larger number of patients.

